# Mass‐flowering crops dilute pollinator abundance in agricultural landscapes across Europe

**DOI:** 10.1111/ele.12657

**Published:** 2016-08-17

**Authors:** Andrea Holzschuh, Matteo Dainese, Juan P. González‐Varo, Sonja Mudri‐Stojnić, Verena Riedinger, Maj Rundlöf, Jeroen Scheper, Jennifer B. Wickens, Victoria J. Wickens, Riccardo Bommarco, David Kleijn, Simon G. Potts, Stuart P. M. Roberts, Henrik G. Smith, Montserrat Vilà, Ante Vujić, Ingolf Steffan‐Dewenter

**Affiliations:** ^1^ Department of Animal Ecology and Tropical BiologyBiocenterUniversity of WürzburgAm Hubland97074WürzburgGermany; ^2^ Estación Biológica de Doñana (EBD‐CSIC)Avda. Américo Vespucio s/n, Isla de la Cartuja41092SevillaSpain; ^3^ Department of Biology and EcologyFaculty of SciencesUniversity of Novi SadTrg Dositeja Obradovića 221000Novi SadSerbia; ^4^ Department of BiologyLund University223 62LundSweden; ^5^ AlterraAnimal Ecology Team6700 AAWageningenThe Netherlands; ^6^ Centre for Agri‐Environmental ResearchSchool of Agriculture, Policy and DevelopmentUniversity of ReadingReadingRG6 6ARUK; ^7^ Department of EcologySwedish University of Agricultural Sciences75007UppsalaSweden; ^8^ Plant Ecology and Nature Conservation GroupWageningen UniversityDroevendaalsesteeg 3a6708PBWageningenThe Netherlands; ^9^ Centre for Environmental and Climate ResearchLund University223 62LundSweden

**Keywords:** Agricultural intensification, agri‐environment schemes, biofuels, crop pollination, ecosystem services, field boundaries, landscape composition, non‐crop habitats, semi‐natural habitats, spillover

## Abstract

Mass‐flowering crops (MFCs) are increasingly cultivated and might influence pollinator communities in MFC fields and nearby semi‐natural habitats (SNHs). Across six European regions and 2 years, we assessed how landscape‐scale cover of MFCs affected pollinator densities in 408 MFC fields and adjacent SNHs. In MFC fields, densities of bumblebees, solitary bees, managed honeybees and hoverflies were negatively related to the cover of MFCs in the landscape. In SNHs, densities of bumblebees declined with increasing cover of MFCs but densities of honeybees increased. The densities of all pollinators were generally unrelated to the cover of SNHs in the landscape. Although MFC fields apparently attracted pollinators from SNHs, in landscapes with large areas of MFCs they became diluted. The resulting lower densities might negatively affect yields of pollinator‐dependent crops and the reproductive success of wild plants. An expansion of MFCs needs to be accompanied by pollinator‐supporting practices in agricultural landscapes.

## Introduction

The decline of wild pollinators has raised serious concerns about the future of crop and wild plant pollination (Biesmeijer *et al*. 2006; Garibaldi *et al*. 2011; González‐Varo *et al*. 2013). Even if increased use of managed pollinators (e.g. the honeybee, *Apis mellifera* L.) can provide some insurance, they cannot fully replace the loss of wild pollinators, since the yields of many crops relate more to the densities of wild pollinators than to that of honeybees (Garibaldi *et al*. 2013). During recent decades, agricultural intensification has led to yield increases in pollinator‐independent crops, but not in insect‐pollinated crops (Aizen *et al*. 2009). A possible reason is that intensification has caused pollinator declines, with negative consequences for crop pollination and yields (Deguines *et al*. 2014). Another, alternative and untested hypothesis, is that the current expansion of mass‐flowering crops (MFCs) could – according to the ‘landscape‐moderated concentration and dilution hypothesis’ proposed by Tscharntke *et al*. (2012) – cause a dilution of pollinator density with a negative effect on pollination services but without necessarily reducing pollinator population size. Between 1961 and 2006, the global cropping area has increased by 23%, of which 18–35% has been devoted to MFCs in the developed world and 23–33% in the developing world (Aizen *et al*. 2008), partially driven by increasing demands for and subsidies to biofuels (Breeze *et al*. 2014).

Although different studies have shown a positive effect of MFCs on pollinator densities (e.g. Westphal *et al*. 2003; Jauker *et al*. 2012a; Holzschuh *et al*. 2013; Rundlöf *et al*. 2014; Riedinger *et al*. 2015), it is not yet known whether MFCs would increase pollinator population growth, or simply transiently attract pollinators to mass bloom, leading to a temporary redistribution of the same populations. It is also not known if this differs between pollinator guilds or different landscape contexts. While there is evidence that solitary bees benefit from oilseed rape fields (Riedinger *et al*. 2015), bumblebees showed contrasting results. For instance the densities of short‐tongued bumblebees, but not that of long‐tongued bumblebees, have been found to increase with increasing cover of oilseed rape fields (Diekötter *et al*. 2010), however, this fails to translate into an increase in the percentage of colonies producing sexual offspring (Westphal *et al*. 2009). In addition, increased cover of MFCs might change the distribution of pollinators within the agricultural landscape [see ‘cross‐habitat spillover hypothesis’ by Tscharntke *et al*. (2012)] and affect pollinator densities in both crop fields (Holzschuh *et al*. 2011; Williams *et al*. 2012) and semi‐natural habitats (SNHs) (Hanley *et al*. 2011; Kovács‐Hostyánszki *et al*. 2013; Persson & Smith 2013). Despite the increasing interest in the effects of MFCs on pollinator populations, it remains unclear how the ongoing expansion of MFCs is changing the distribution of functionally relevant pollinator guilds in crops and SNHs (Schellhorn *et al*. 2015).

**Figure 1:**
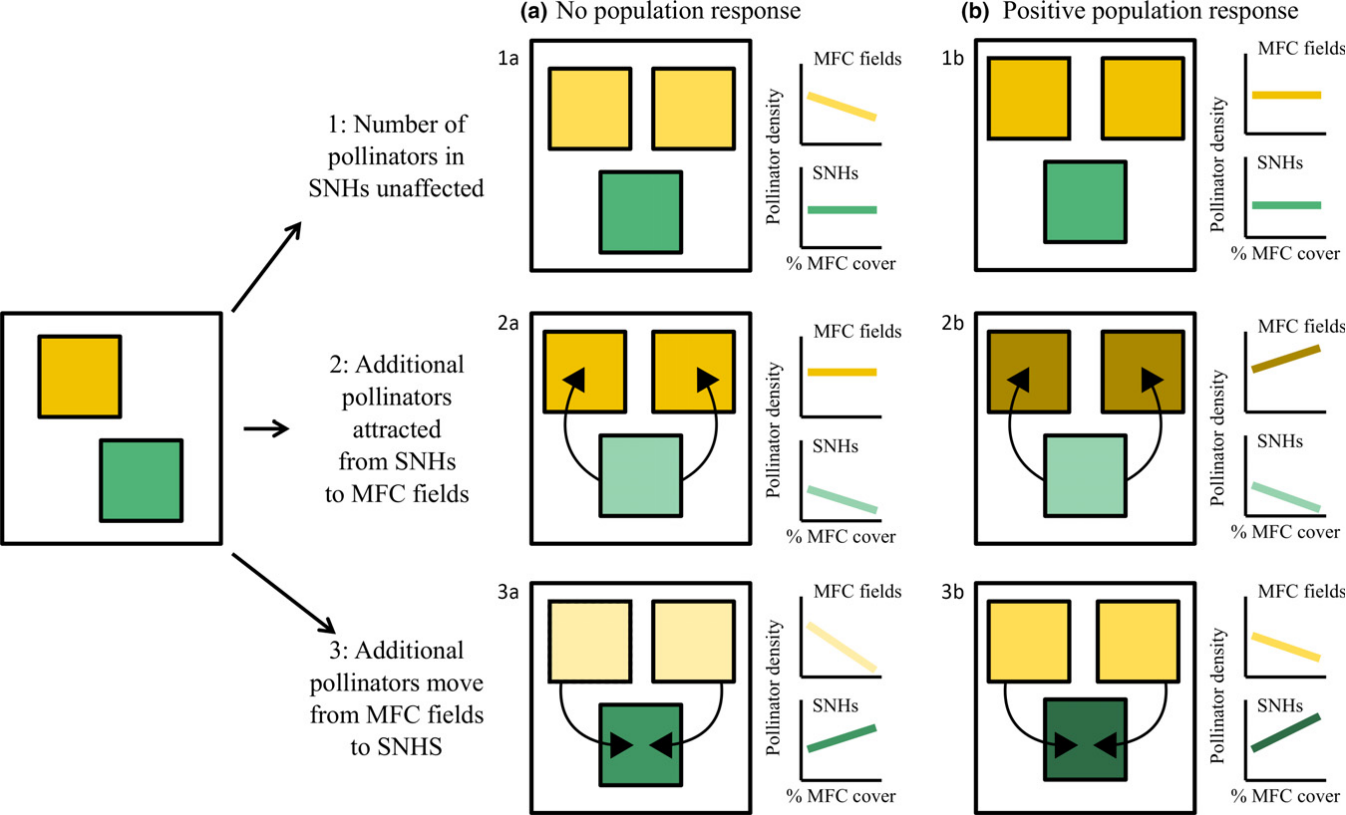
Six hypothetical groups of responses (1a–3b) illustrating the possible consequences of increased cover of mass‐flowering crops (MFCs) on pollinator densities in MFC fields (yellow squares) and in semi‐natural habitats (SNHs; green squares). The response type depends on how an increased cover of MFCs affects the distribution of pollinators in the landscape (responses 1–3; arrows between squares show the expected direction of pollinator flows under increased cover of MFCs) and on whether total population size is unaffected or increased when the cover of MFCs increases (responses a vs. b). Increased pollinator densities are visualised by an increased darkening of shades in the squares (yellow in MFC fields and green in SNHs). The hypothesised relationships between the cover of MFCs and pollinator densities in MFC fields (yellow line) and in SNHs (green line) are shown in graphs.

Here, we assessed how the population densities of different pollinator guilds changed in response to increased cover of MFCs and SNHs. We hypothesised that local pollinator densities in the MFCs and SNHs would show a negative, neutral or positive response to increased cover of MFCs based on six alternative scenarios (Fig. 1). Pollinator population growth may be constrained by the availability of nesting resources (Potts *et al*. 2005; Steffan‐Dewenter & Schiele 2008; but see Roulston & Goodell 2011) and food resources throughout the season (Westphal *et al*. 2009; Williams *et al*. 2012; Rundlöf *et al*. 2014). If nesting and forage resources are limiting – which is probable in landscapes with a low cover of SNHs that usually provide these resources – pollinator populations cannot grow proportionally with the increasing cover of MFCs. In this situation, population size does not change, but local pollinator densities decrease with increased cover of MFCs due to dilution of individuals in the landscape (responses 1a, 2a and 3a in Fig. 1). In contrast, if nesting resources and floral resources throughout the season do not limit population size, a situation we assume to occur in landscapes with a high cover of SNHs, the population size of pollinators that strongly depends on the availability of floral resources during the mass bloom increases with expanding cover of MFCs (Riedinger *et al*. 2015) (responses 1b, 2b and 3b in Fig 1). However, local pollinator densities remain constant or decrease if this population increase is equal or smaller than the increase in MFCs in the landscape. For pollen foragers, the preference of diverse floral resources in SNHs can result in a redistribution of pollinators from MFC fields to SNHs (Danner *et al*. 2016).

The response type depends not only on whether total population size is unaffected or increased when MFC cover increases (responses a vs. b) but also, on how an increased MFC cover affects the distribution of pollinators in the landscape (responses 1 to 3 in Fig. 1) in relation to neighbouring SNHs, as follows:

1. *Pollinator densities remain unaffected in the SNHs irrespective of MFC cover* (responses 1a and 1b). In the MFC fields, we assume that in the absence of a population response there will be a decrease in pollinator densities with increased cover of MFCs due to dilution of individuals in the landscape (response 1a) (Holzschuh *et al*. 2011; Riedinger *et al*. 2014). In contrast, cover of MFCs will not affect pollinator densities if the population size increases in parallel with an increase in MFC cover (response 1b). An intermediate positive effect of MFC would also be plausible if population size increase can only partly compensate for dilution effects.
2. *Pollinator densities decline in SNHs due to a greater attraction of increased MFC cover* (responses 2a and 2b). This happens when MFCs and nesting sites or larval habitats are in close proximity, and SNHs are of lower quality, e.g. low floral density (Kovács‐Hostyánszki *et al*. 2013). In the MFCs, we assume that in the absence of a population response pollinator densities will remain unaffected due to an increased attraction of pollinators to the MFCs (response 2a). In contrast, a combination of population response and pollinator supplementation from SNHs will lead to increased pollinator densities in the MFC fields (response 2b). Generally, the fewer additional pollinators are attracted from SNHs to the MFC, the more similar responses 2a and 2b are to responses 1a and 1b respectively.
3. *Pollinators move from MFCs to neighbouring SNHs, which provide less abundant but more diverse floral resources* (Hanley *et al*. 2011) (responses 3a and 3b). The proportion of pollinators moving from MFCs to SNHs increases with increasing cover of MFCs and decreasing distances between habitat types. The more pollinators relocating from MFCs to SNHs, the more strongly densities will decrease in MFC fields and increase in SNHs. We assume a greater probability of such movements to high‐quality SNHs than to low‐quality SNHs. In a no population response situation, a steeper decline in crop pollinator densities and a smaller increase in SNHs is expected (response 3a) with increasing cover of MFCs. However, given a positive population response, pollinator density declines will be less pronounced in MFC fields and increase greater in SNHs (response 3b).

Here, we test these alternative scenarios for the first time in a large‐scale study. Densities of foraging pollinators (bumblebees, solitary bees, managed honeybees and hoverflies) were recorded in six regions across Europe over a 2‐year period (2011–2012) in 408 focal MFC fields and SNHs (linear and patch) embedded in 93 study areas varying in the cover of MFCs and SNHs. The data allowed us to test the predictions described in Fig. 1, on pollinator densities and distribution across the landscape. Also, the data allowed us to assess differences among pollinator guilds, among landscapes differing in the cover of SNHs, and SNHs differing in quality (linear field boundaries vs. semi‐natural patches). Our study is the first that allows an assessment of some of the risks and benefits for pollination functions arising from the current expansion of pollinator‐dependent MFCs across Europe.

## Material and Methods

The study was conducted in six regions in Germany, the Netherlands, Serbia, Spain, Sweden and the UK (i.e. one region per country) in 2011 and 2012. In each region, we selected 14–16 sites where half were characterised by high relative cover of the dominant MFC for the region, and half showed low or no coverage. The dominant pollinator‐dependent mass‐flowering crop (MFC) was: oilseed rape (*Brassica napus* L.) in Sweden, the UK, the Netherlands and Germany; sunflower (*Helianthus annuus* L.) in Serbia; and orange (*Citrus × sinensis* L.) in Spain. Within each site, three focal habitat types were selected (but see exceptions in Table 1): (1) the dominant MFC, (2) a linear semi‐natural habitat represented by an uncultivated field boundary with a minimum width of 1 m, and (3) a nonlinear semi‐natural habitat patch (SNH) such as grassland or forest which was selected on the basis of its quality as pollinator habitat (Table 1). In Serbia and Spain, no substantial linear semi‐natural habitats were present and so field boundaries were not included. In each region, the distance between the three habitat types within the same site aimed for 100–500 m, whereas the distance between the same habitat type (between sites) was at least 2000 m. Due to crop rotation in oilseed and sunflower, new MFC fields were locally reselected in 2012. SNHs were the same in both years except seven sites that could not be sampled in 2012, as they were either inaccessible or highly disturbed. New sites replaced three of these sites. In the UK, the study was conducted in 2012 and 2013 but only data from 2012 was included in the analysis. In total, 80 sites with 184 focal habitats were surveyed in 2011, and 93 sites with 224 focal habitats in 2012 (Table 1).

**Table 1:** Overview of three focal habitat types and the number of replicates in the six study regions (*n *= number of replicates in 2011/in 2012)

Country	MFC fields (total *n *=* *56/68)	Field boundaries (total *n *=* *48/64)	Semi‐natural habitats (total *n *=* *80/92)
Sweden	Oilseed rape fields (*n *=* *16/16)	Semi‐permanent field margins (*n *=* *16/16)	Uncultivated grasslands (*n *=* *16/16)
UK	Oilseed rape fields (*n *=* *0/16)	Permanent field margins (*n *=* *0/16)	Uncultivated species‐rich grasslands (*n *=* *0/15)
Netherlands	Oilseed rape fields (*n *=* *8/6^a^)	Permanent field margins (*n *=* *16/16)	Forest edges (*n *=* *16/16)
Germany	Oilseed rape fields (*n *=* *16/16)	Permanent field margins (*n *=* *16/16)	Uncultivated species‐rich grasslands (*n *=* *16/16)
Serbia	Sunflower fields (*n *=* *8/7^a^)	(not available)	Uncultivated species‐rich grasslands (*n *=* *16/15)
Spain	Orange orchards (*n *=* *8/7^a^)	(not available)	Understory of *Pinus pinea* woodlands (*n *=* *16/14)

a Number of sites is lower than 16, because in half of the areas, no mass‐flowering crops were grown.

Around each focal habitat, the landscape was characterised within a 1000 m radius from the habitat edge. This radius was chosen because, for all pollinator guilds relevant in the current studies, it has been shown that most foraging flights are within this distance (Steffan‐Dewenter & Kuhn 2003; Holzschuh *et al*. 2007, 2011; Rundlöf *et al*. 2008; Haenke *et al*. 2014). In all landscapes, the percentage cover of SNHs and the regionally dominant MFC were calculated annually from ground‐truthed maps using ArcGIS (hereafter ‘cover of MFCs’ and ‘cover of SNHs’) (see Table S1 in Supporting Information). Habitats were classified as SNHs if they provided bee nesting sites and floral resources and were considered to be valuable for a relatively high number of bee and hoverfly species (see Appendix S1). Collinearity between the cover of MFCs and SNHs surrounding each site was controlled by each region and each year per region separately. The two variables were uncorrelated with the exception of Spain (Pearson correlations conducted separately for landscapes around MFC fields, field boundaries and SNHs: all |*r*| < 0.48, all *P *>* *0.05, Spain: − 0.77 < *r *< − 0.67, 0.001 < *P *<* *0.074). We repeated the analysis without the Spain data included and ensured that this did not affect our results (analysis not show).

In 2011 and 2012, flower‐visiting bees (Hymenoptera: Apiformes) and hoverflies (Diptera: Syrphidae) as main oilseed rape pollinators (Jauker *et al*. 2012b) were recorded by a slowly walking observer, during two survey rounds at peak flowering of the MFC within two transects (150 m long and 1 m wide) for 15 minu per transect per site per round (see Appendix S2). Other, minor flower‐visiting insect groups, were not included in the study. We refer to all wild bee species other than bumblebees as ‘solitary bees’, although some halictid bee species are primitively eusocial. Flower cover of plants flowering within the two 150 m² transects during the pollinator survey was estimated using quadrats (see Table S2). Flower cover and densities of bumblebees, solitary bees, managed honeybees and hoverflies were averaged over the two transects and the two survey rounds per site.

### Statistical analysis

All statistical analysis was performed using R. We used linear mixed‐effect models with a normal error distribution using the ‘lme4’ package to test whether the cover of MFCs affected pollinator densities and whether the effects were consistent across landscapes varying in their cover of SNHs. Separate models were run for the three focal habitat types. Response variables were the four guilds, i.e. densities of bumblebees, solitary bees, honeybees and hoverflies were analysed in separate models (Holzschuh *et al*. 2016). Predictors included the cover of MFCs, the cover of SNHs and their interaction. The co‐variable local flower cover was included in models for SNHs and field boundaries. All response variables were ln (*x *+* *1) transformed to meet the normality and homoscedasticity assumptions of the models, as well as the predictors to linearise their relationships with the response variables.

To control for interannual and among regions variability in pollinator densities, we included region identity and year as crossed random factors. We tested three random structures differing in the complexity of their formulation by likelihood ratio tests: (1) random intercept for region and year, (2) random intercept for region and year and region‐level slope for the landscape effect (MFCs cover) and (3) random intercept for region and year and region and year level slopes for the landscape effect (MFCs cover). In this way, we tested whether the resulting effects were consistent across regions and years. We first built full models and then simplified them by removing one‐by‐one the non‐significant fixed terms, while respecting marginality. *F* and *P* values were interpreted using Satterthwaite's approximations to determine denominator degrees of freedom in package ‘lmerTest’. We assessed the robustness of parameter estimates from the 95% bootstrapped confidence intervals (CIs, *n *=* *1000). In addition, all models were re‐calculated using standardised ln‐transformed response variables and continuous predictors (i.e. *z*‐scores) to account for possible differences in the range of MFC and SNH cover across the regions and in species abundance across years and regions. All results were consistent with the two approaches (unstandardised vs. standardised variables; see results of models fitted with standardised variables in Table S3). Further details for statistical packages used for the analyses are given in Appendix S3.

## Results

Overall, across the six countries and two years we recorded 22 887 pollinators (1677 bumblebees, 3573 solitary bees, 13 400 honeybees and 4237 hoverflies) (see Fig. S1).

**Figure 2:**
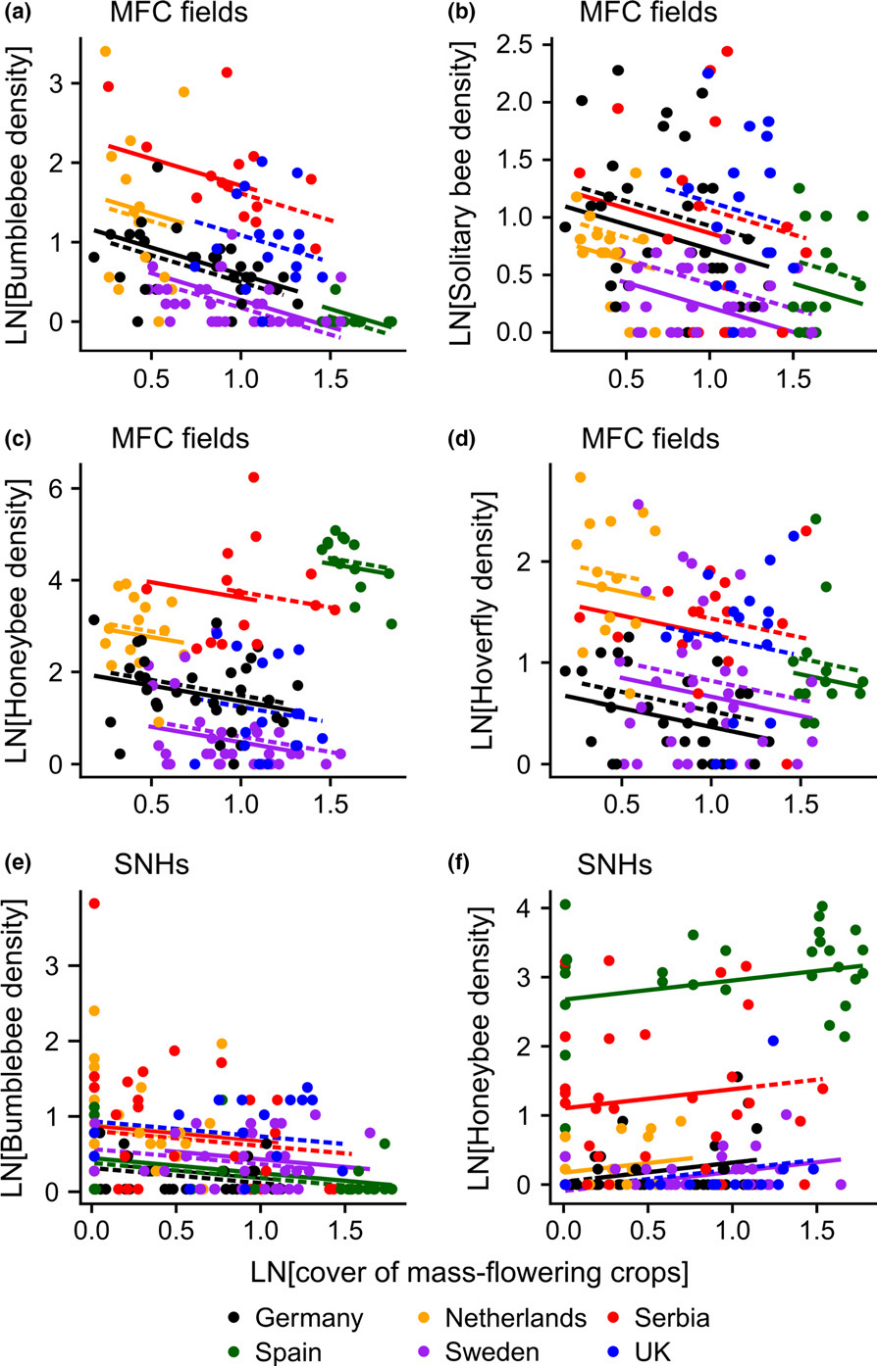
Relationships between the cover of MFCs (% in 1 km radius) and densities of (a) bumblebees, (b) solitary bees, (c) honeybees and (d) hoverflies in MFC fields, and densities of (e) bumblebees and (f) honeybees in semi‐natural habitat patches (SNHs). Colour indicates the different study regions. The fitted lines are linear mixed model estimates for each region and year (2011, solid lines; 2012, dashed lines).

In MFCs, densities of all pollinator guilds declined with increasing cover of MFCs in the surrounding landscape within a 1 km radius (Fig. 2a–d). These relationships did not differ among regions (random slope non‐significant in all models). Each doubling of the MFC cover reduced densities of bumblebees, solitary bees, honeybees and hoverflies in MFCs by 15, 10, 15 and 7% respectively. For example comparing the MFC site with the lowest to the highest cover of MFCs within each region, our models estimated an average decline (mean ± SE, hereafter) of both total bumblebee and honeybee densities by 35 ± 5% (range of decline: 14–51%).

In SNHs, bumblebee densities declined with increasing cover of MFCs (Table 2, Fig. 2e). Each doubling of the cover of MFCs reduced densities of bumblebees by 5% in SNHs. Comparing the SNH sites with the lowest to the highest cover of MFCs within each region, the model estimated an average decline of bumblebee densities by 21 ± 2% (range of decline: 11–27%). In contrast to bumblebees, honeybee densities in SNHs increased with increasing cover of MFCs (9% per doubling of crop cover; Fig. 2f). Comparing the landscapes with the lowest and the highest cover of MFCs within each region, the model predicted an average increase in honeybee densities in SNHs by 40 ± 6% within regions (range of increase: 19–62%). Again, these two relationships were consistent across regions (random slope non‐significant in both models). Densities of solitary bees and hoverflies in SNHs were instead unrelated to the cover of MFCs.

**Table 2:** Results of linear mixed effects models relating densities of bumblebees, solitary bees, honeybees and hoverflies in MFCfields, fieldboundaries and semi‐natural habitats to the predictors cover of the mass‐floweringcrop in a 1 km radius (MFC), cover of semi‐natural habitats in a 1 km radius (SNH) and local flower cover (FC).Model estimate (β) and 95% confidence intervals (CIs)are reported. Only significant main effects and interactions are shown

	MFC fields		Field boundaries		Semi‐natural habitats	
	β (95% CIs)	*P*	β (95% CIs)	*P*	β (95% CIs)	*P*
*Bumblebees*						
MFC	− 0.29(− 0.45, − 0.16)	< 0.001	0.23(− 0.02, 0.49)	0.081	− 0.08(− 0.14, − 0.01)	0.022
SNH	–	–	0.17(− 0.16, 0.79)	0.107	–	–
FC	–	–	0.14 (0.02, 0.39)	0.008	–	–
MFC:SNH	–	–	− 0.16 (− 0.28, − 0.05)	0.007	–	–
*Solitary bees*						
MFC	− 0.20 (− 0.34, − 0.05)	0.006	–	–	–	–
SNH	–	–	0.19 (0.04, 0.34)	0.024	–	–
FC	–	–	0.16 (0.03, 0.30)	0.019	0.36(0.16, 0.58)	< 0.001
*Honeybees*						
MFC	−0.29(−0.54, − 0.03)	0.024	–	–	0.12 (0.03, 0.21)	0.009
*Hoverflies*						
MFC	− 0.18(− 0.34, − 0.02)	0.030	–	–	–	–
FC	–	–	0.33 (0.15, 0.51)	< 0.001	0.51(0.28, 0.74)	< 0.001

**Figure 3:**
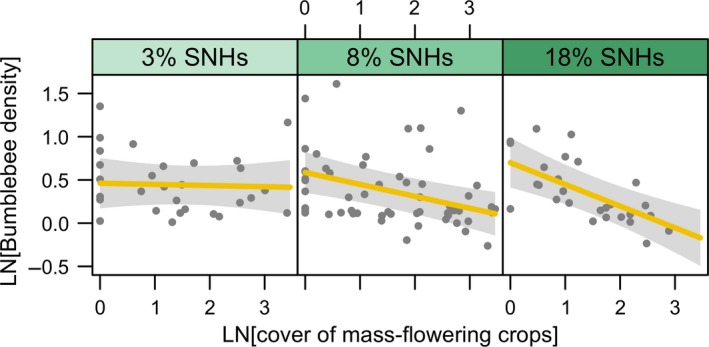
The interaction between the cover of mass‐flowering crops (MFCs) and semi‐natural habitats (SNHs) in the landscape on bumblebee densities in field boundaries. Panels are ranked from left to right according to increasing proportion of SNH cover in a radius of 1 km surrounding each field boundary. The fitted lines are linear mixed model estimates from the model described in Table 2.

In field boundaries, density of bumblebees was explained by an interaction between the covers of MFCs and SNHs (Table 2). The relationship between bumblebee density and the cover of MFCs was negative in the most heterogeneous landscapes (high cover of SNHs), was weak at intermediate levels, whereas disappeared in simpler landscapes (Fig. 3). Densities of solitary bees, honeybees and hoverflies in field boundaries were unrelated to the cover of MFCs.

For solitary bees, honeybees and hoverflies, the cover of SNHs did not show any (additive or interactive) effect on pollinator densities in either MFCs or SNHs (Table 2). The same result was found for honeybees and hoverflies in field boundaries, while the cover of SNHs showed an additive effect for solitary bees (Table 2). In field boundaries, densities of bumblebees, solitary bees and hoverflies were positively related to local flower cover, while in SNHs this relationship was only found for solitary bees and hoverflies (Table 2).

## Discussion

We found a consistent negative relationship between the cover of pollinator‐dependent mass‐flowering crops (MFCs) and pollinator densities in MFC fields across Europe. This is the first evidence that an increased cultivation of MFCs decreases densities of bumblebees, solitary bees, honeybees and hoverflies in the MFC fields across regions. In semi‐natural habitat patches (SNHs) bumblebee densities also declined, whereas densities of honeybees increased, and densities of solitary bees and hoverflies were not related to increasing cover of MFCs. In field boundaries, the cover of SNHs in the landscape modulated the effect of MFC cover only on bumblebee densities.

For bumblebees, solitary bees, honeybees and hoverflies in MFC fields, our results suggest that none of these pollinator groups increased their population size in the landscape proportionally with the increased floral resource availability in the landscape supplied by MFCs (responses 1a–3a; Fig. 1). While pollinators may be visiting MFCs, these floral resources may be of insufficient quality to enhance the reproduction of pollinators proportionally to the amount of resources. Indeed, the type and diversity of floral resources available might affect individual bee health and colony fitness (Goulson *et al*. 2015). This can be the case, for example if the composition of amino acid or other nutritional components of the crop pollen is unfavourable (Roulston & Cane 2000), if crop pollen components are toxic (Sedivy *et al*. 2011) or if pollinators are pollen specialists and forage in the crop for nectar only. It could also be that factors other than the floral resources provided by MFCs limit the growth of pollinator populations. For instance the availability of nest sites (Potts *et al*. 2005; Steffan‐Dewenter & Schiele 2008; but see Roulston & Goodell 2011) and the continuity of floral resources after the cessation of crop mass‐flowering can limit pollinator densities (Westphal *et al*. 2009; Williams *et al*. 2012; Rundlöf *et al*. 2014; Riedinger *et al*. 2015). Natural enemies can also limit pollinator population growth although there are few examples showing this (Rosenheim 1990; Roulston & Goodell 2011). In contrast to bees, hoverflies do not depend on nesting sites, but, because their larvae require other food resources (larvae can be saprotrophs, eating decaying plant and animal matter in the soil; or herbivores on different parts of plants, or insectivores predating on aphids and other plant‐sucking insect) (Gilbert 1993), only adult hoverflies can benefit from crop floral resources. The total number of hoverflies in the landscape can therefore only positively respond to increased mass‐flowering crop cover if the availability of larval food is not limiting.

For honeybees, we not only found a decline in densities in the MFC fields with increasing cover of MFCs, but also an increase in densities in the SNHs. These relationships suggest that an increase in cover of MFCs enhances the number of visits to SNHs (responses 3a and 3b; Fig. 1). This increase may have resulted from a redistribution of honeybees from the flower‐dense MFC fields to the less dense, but more diverse floral resources in SNHs (Hanley *et al*. 2011; Tscharntke *et al*. 2012; Haenke *et al*. 2014), a phenomenon that has received little attention (Blitzer *et al*. 2012). In contrast to wild bees, honeybees prefer to exploit mass‐resources for nectar collection (Stanley & Stout 2014; Requier *et al*. 2015) leading to comparably high densities in MFC fields. Nevertheless, collecting pollen from a wide diversity of plants to improve the diet composition (Requier *et al*. 2015) could explain the high honeybee densities in SNHs in landscapes with high cover of MFCs (Danner *et al*. 2016). Our results show that the redistribution of honeybees to SNHs increases when cover of MFCs increases, suggesting that such relocation rather occurs when distances between habitats decline. In addition, such redistribution of honeybees to SNHs could be modulated by the relative attractiveness of floral resources in MFCs and SNHs, which depends on crop types, SNHs quality and seasonal phenology of wild flower resources (Danner *et al*. 2016). The absence of similar results in field boundaries can be explained by their low habitat quality (i.e. low availability of floral resources) compared to larger SNHs, which was not sufficient to attract foraging honeybees. Whilst total honeybee numbers in the landscape can be enhanced by increasing colony numbers, in most European countries the area of pollinator‐dependent crops is currently increasing more than the supply of honeybee colonies (Breeze *et al*. 2014). As a consequence, if the active placement of honeybee hives is not proportional to the increased cover of MFCs, we can observe a decrease in honeybee densities in MFC fields with increased cover of MFCs. Further studies are needed to understand whether an increase in honeybee colony densities in combination with increased cover of MFCs drives honeybee relocation to SNHs and increase competition between wild and honeybees (Hudewenz & Klein 2013).

Bumblebees were the only pollinator group whose densities decreased with increasing cover of MFCs in MFC fields, SNHs and field boundaries. These relationships are consistent with the assumption that more pollinators are attracted from SNHs to MFCs when the cover of MFCs increased (response 2a; Fig. 1). Our results provide a possible cause for the previous finding that the reproduction of a bumblebee‐pollinated wild plant species in semi‐natural grasslands is reduced in landscapes with high cover of MFCs (Holzschuh *et al*. 2011). An alternative explanation for decreasing bumblebee densities in SNHs with increasing cover of MFCs is that bumblebees avoided the increased honeybee densities at these sites and reduced interspecific competition by visiting MFC fields instead. Although the redistributed bumblebees might have mitigated dilution effects occurring when the cover of MFCs is high, their numbers were not large enough to enhance bumblebee densities in MFC fields up to the densities recorded in landscapes with a low cover of MFCs. In contrast to a regional study suggesting that crop fields do not pull bumblebees from high‐quality habitats (Kovács‐Hostyánszki *et al*. 2013), our data show that MFCs attracted bumblebees from both field boundaries and high‐quality SNHs. Notably bumblebee densities in field boundaries strongly decreased with increasing cover of MFCs only in more heterogeneous landscapes (high cover of SNHs). This suggests that bumblebees concentrated at field boundaries if the cover of other, flower‐rich SNHs was low, a pattern that has been previously found for trap‐nesting bees (Diekötter *et al*. 2014).

Interestingly, cover of MFCs did not affect densities of solitary bees and hoverflies in SNHs, probably because the majority of solitary bee and hoverfly species occurring in these SNHs are not attracted by MFCs, making it difficult to detect a potential effect of MFCs (Meyer *et al*. 2009). Further studies focusing on the trait‐specificity of spillover effects, for example the role of pollen resource specialisation, sociality, body size and foraging distances (Bommarco *et al*. 2010; Jauker *et al*. 2012a), might help to disentangle differences in pollinator responses to increased cover of MFCs.

In contrast to our expectation, a high cover of SNHs did not diminish the decline of pollinator densities with increasing cover of MFCs. Other studies have shown that the availability of high‐quality habitats, providing nesting sites and floral resources throughout the season, strongly limit wild bee densities (Garibaldi *et al*. 2011; Kennedy *et al*. 2013). In our study, the importance of SNHs was marginal compared to the dilution effect of MFCs. This could be because it was a predetermined criterion of our study design that at least one high‐quality SNH was present in 1 km radius around the study crop fields. An alternative explanation is that our study design aimed to maximise the gradient in cover of MFCs while keeping the cover of SNHs uncorrelated; however, the range was larger for SNHs than for MFCs in all countries except Sweden. Nevertheless, as we found stronger effects of MFC cover than of SNH cover for all pollinator groups in MFC fields, this emphasises the strength of dilution effects of MFCs on pollinator densities in agricultural landscapes. Although we are observing a rapid increase in the cover of MFCs across European landscapes, pollinator populations have no clear advantage of this land‐use change, indicating that MFCs are not increasing pollinator population size.

The decline of pollinator densities might have direct consequences for the pollination services provided to both crop and wild plants. Declines in wild pollinator densities are paralleled by declines in fruit and seed set in 41 pollinator‐dependent crops assessed in a global synthesis (Garibaldi *et al*. 2013). Moreover, recent studies highlight that insect pollination can additionally enhance yield quality in various European crops, for example the oil content of rapeseed and the commercial grade of fruits (Bommarco *et al*. 2012; Bartomeus *et al*. 2014; Klatt *et al*. 2014). Our findings suggest that managed honeybees currently are not able to compensate for declining densities of wild pollinators in crop fields. This is in accordance with a previous finding that the demand for honeybee colonies required for crop pollination currently surpasses the supply in the majority of European countries (Breeze *et al*. 2014). Therefore, the expansion of MFCs needs to be accompanied by pollinator‐supporting practices in agricultural landscapes (Garibaldi *et al*. 2014; Scheper *et al*. 2015). From a conservation perspective, the decline of wild pollinators is most critical in SNHs, because it might directly translate into a decline in the reproductive success of pollinator‐dependent wild plants (Biesmeijer *et al*. 2006; Holzschuh *et al*. 2011; Clough *et al*. 2014). Furthermore, the increased densities of honeybees in landscapes with high MFCs might enhance resource competition for specialised and endangered wild bee populations.

To optimise pollination services in crop fields, we need pollinator populations capable of increasing proportionally with the agriculturally driven demand for pollinators. Factors that limit the growth of pollinator populations in response to expansion of MFCs should be addressed in future studies and, thus, targeted by management policies. There is also a need for greater knowledge about the temporal dynamics of pollinator populations as the cover of insect‐pollinated crops can widely vary from year to year due to the fluctuation of crop prices, agricultural subsidies and crop rotation (Riedinger *et al*. 2015). Furthermore, negative impacts of agricultural intensification, particularly pesticides, on pollinators need to be reduced (Rundlöf *et al*. 2015), otherwise pollinator populations may not be able to fully benefit from enhanced resources provided by MFCs and agri‐environment schemes.

## Authorship

AH, RB, DK, MR, SGP, HGS, AV, MV and ISD developed the experimental design; JPG, SMS, VR, MR, JS, JBW and VJW collected data; AH and MD analysed the data and AH wrote the first draft with input from MD and ISD. All authors contributed to revisions of the manuscript.
