# Supplementary material for: Mass‐flowering crops dilute pollinator abundance in agricultural landscapes across Europe
Source: Ecol Lett. 2016 Aug 17;19(10):1228–36. doi: 10.1111/ele.12657 (PMC5031195; doi:10.1111/ele.12657)
Supplement: Supplementary file 1 [file ELE-19-1228-s001.pdf]

Supplementary Information

Appendix S1. List of semi-natural habitats mapped in the six study regions.

Germany: uncultivated or non-intensively used grasslands, forest edges (10m wide forest border), hedges, orchard meadows, gardens outside settlements, annual flower-rich fallows, sown flower strips.

The Netherlands: uncultivated or non-intensively used grasslands, hedges, forest edges (10m wide forest border), heathland, extensive flower-rich cereal fields.

Serbia: non-intensively used grasslands, wet meadows, permanent fallows, hedges, gardens outside settlements.

Spain: hedges, forest, fallows, olive groves, shrubland, marshland, Mediterranean savanna, riparian shrub vegetation.

Sweden: permanent, agrochemical-free grasslands used for grazing or traditional late-season mowing, ”biodiversity” fallows, field borders (estimated from the length of field borders and with a width of 1 m), semi-permanent grassy-herbaceous fallows along waterways, hedges.

UK: uncultivated or non-intensively used grasslands, permanent flower-rich fallows, hedges, forests, gardens outside settlements.

Appendix S2. Pollinator monitoring.

In mass-flowering crop fields, one transect parallel to the field edge along the outer crop row and a second transect in the field interior at least 20 m away from all field edges. The two transects in field boundaries and semi-natural habitats were running through the most flower-rich parts of the site and varied in location between survey rounds (Westphal et al. 2008). In each site, two survey rounds – one in the morning and one in the afternoon – were conducted on different days during the mass-flowering period of the crop (total recording time per site: 2 [transects] × 15 min × 2 [rounds] = 60 min). Pollinators were recorded between 09:00 and 18:00 h under standardised weather conditions (>17 °C, no rain, low or no cloud cover, low wind speeds). Specimens that could not be identified in the field were brought to the lab for identification.

Reference

Westphal, C., Bommarco, R., Carre, G., Lamborn, E., Morison, N., Petanidou, T., *et al.* (2008). Measuring bee diversity in different European habitats and biogeographical regions. *Ecol. Monogr.*, 78, 653–671.

Appendix S3. Statistical packages used for the analyses.

All statistical analysis were performed using R version 3.2.2 (R Core Team 2015). Linear mixed-effect models were fit using the 'lme4' package (Bates *et al*. 2015). *F* and *P* values in linear mixed-effect models were interpreted using Satterthwaite's approximations to determine denominator degrees of freedom in package 'lmerTest' (Kuznetsova *et al*. 2016).

References

Bates, D., Maechler, M., Bolker, B. & Walker, S. (2015). Fitting linear mixed-effects models using lme4. *J. Stat. Software*, 67(1), 1-48.

Kuznetsova, A., Brockhoff, P.B. & Christensen, R.H.B. (2016). lmerTest: Tests in linear mixed effects models. R package version 2.0-30.

R Core Team (2015). R: A language and environment for statistical computing. R Foundation for Statistical Computing, Vienna, Austria. URL https://www.R-project.org/.

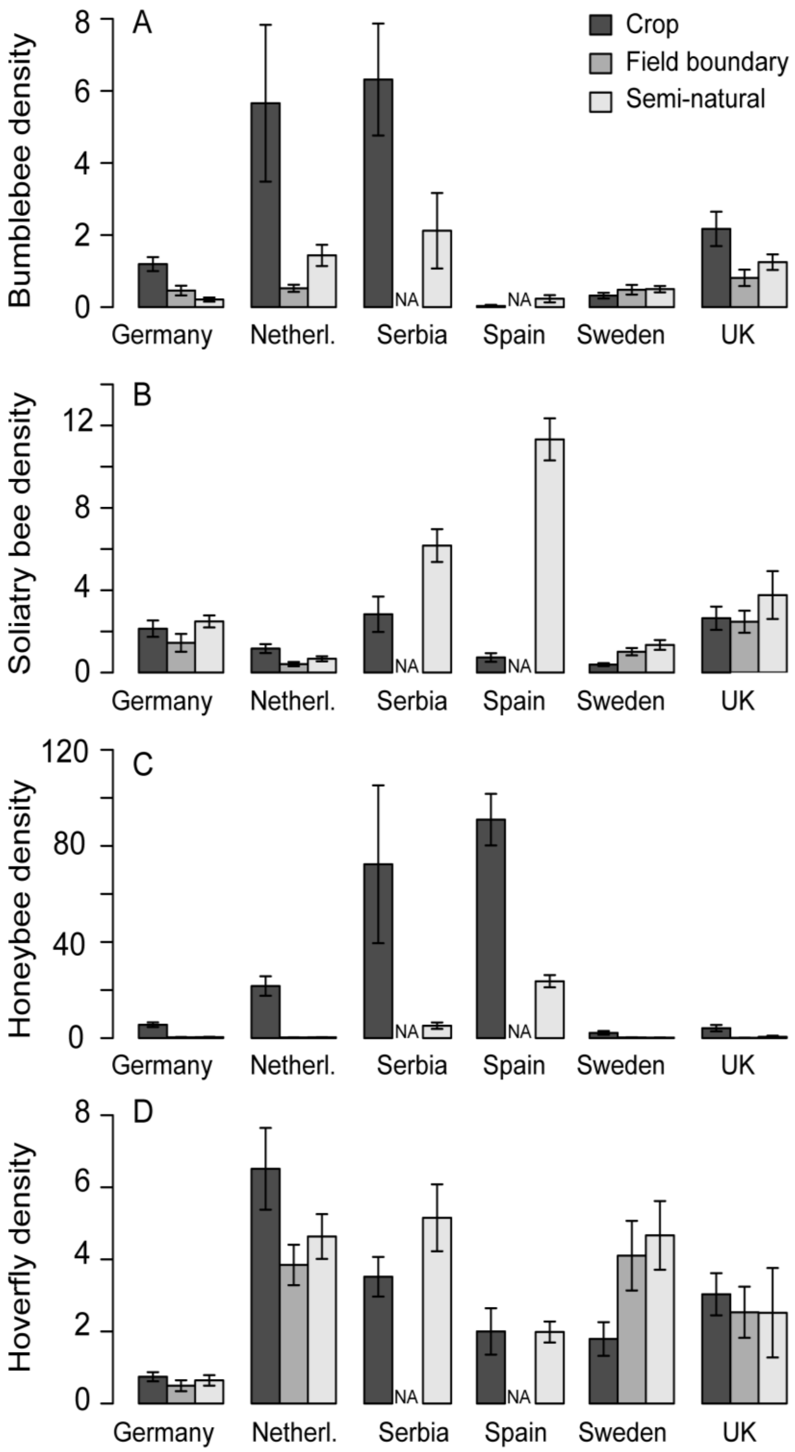

Figure S1. Mean densities (±SE) of pollinators per 150 m² and 15 min in Germany, the Netherlands, Serbia, Spain, Sweden and UK subdivided by pollinator group (panels A-D) and habitat type (pollinator-dependent crop, field boundary, semi-natural habitat). No data are available for field boundaries in Serbia and Spain (NA).

Table S1. Percentage cover of pollinator-dependent crops and semi-natural habitats in a 1 km radius around the study sites (crop fields, field boundaries and semi-natural habitats) in 2011 and 2012. Means ± SE (min - max) are noted.

	2011		2012	
Cover of pollinator-dependent crops				
- crop fields				
Germany	7.05 ± 1.42	(0.51 - 19.80)	5.71 ± 1.06	(0.87 - 16.30)
Netherlands	1.75 ± 0.35	(0.75 - 3.80)	1.87 ± 0.37	(0.83 - 3.11)
Serbia	6.20 ± 1.29	(0.82 - 11.31)	16.88 ± 3.81	(7.35 - 32.53)
Spain	39.13 ± 4.61	(27.40 - 68.56)	40.85 ± 4.76	(29.63 - 66.21)
Sweden	10.88 ± 2.19	(2.04 - 35.29)	10.29 ± 2.31	(2.86 - 35.07)
UK	NA	NA	14.31 ± 1.55	(4.53 - 27.55)
- field boundaries				
Germany	6.62 ± 1.45	(0.54 - 19.21)	4.32 ± 1.01	(0.00 - 12.43)
Netherlands	1.11 ± 0.35	(0.00 - 4.54)	0.70 ± 0.27	(0.00 - 3.72)
Sweden	11.07 ± 2.41	(0.56 - 29.57)	7.89 ± 2.42	(0.00 - 30.88)
UK	NA	NA	11.91 ± 2.08	(0.63 - 28.86)
- semi-natural habitats				
Germany	4.96 ± 1.11	(0.50 - 12.64)	4.59 ± 0.96	(0.08 - 11.81)
Netherlands	1.00 ± 0.35	(0.00 - 4.69)	0.71 ± 0.29	(0.00 - 3.84)
Serbia	3.30 ± 0.97	(0.00 - 11.31)	8.12 ± 2.78	(0.00 - 32.53)
Spain	20.95 ± 5.25	(0.00 - 56.94)	21.08 ± 5.64	(0.00 - 56.94)
Sweden	10.63 ± 2.45	(1.61 - 41.95)	6.98 ± 1.34	(0.00 - 17.75)
UK	NA	NA	9.56 ± 2.07	(0.00 - 28.53)
Cover of semi-natural habitats				
- crop fields				
Germany	11.45 ± 1.66	(1.99 - 24.72)	10.49 ± 1.76	(2.26 - 28.50)
Netherlands	7.02 ± 1.38	(2.38 - 14.72)	6.74 ± 1.56	(2.38 - 12.94)
Serbia	21.13 ± 6.14	(5.87 - 59.57)	15.48 ± 2.77	(5.41 - 28.44)
Spain	35.73 ± 4.89	(18.99 - 60.01)	36.46 ± 4.95	(21.93 - 60.01)
Sweden	9.41 ± 1.00	(3.02 - 19.03)	8.88 ± 1.10	(2.38 - 16.44)
UK	NA	NA	14.39 ± 2.28	(1.80 - 31.49)
- field boundaries				
Germany	11.45 ± 1.58	(1.92 - 25.94)	11.33 ± 1.62	(1.06 - 25.94)
Netherlands	6.66 ± 1.02	(1.01 - 16.18)	6.77 ± 1.07	(1.01 - 16.18)
Sweden	8.36 ± 1.27	(2.79 - 20.89)	8.38 ± 1.27	(2.83 - 20.62)
UK	NA	NA	14.30 ± 1.99	(3.87 - 31.39)
- semi-natural habitats				
Germany	11.92 ± 1.24	(3.72 - 20.48)	11.94 ± 1.23	(3.72 - 20.48)
Netherlands	7.49 ± 1.02	(2.64 - 15.11)	7.49 ± 1.02	(2.64 - 15.11)
Serbia	16.87 ± 1.85	(5.87 - 31.70)	17.14 ± 1.96	(5.41 - 30.69)
Spain	47.35 ± 4.53	(19.66 - 79.65)	48.82 ± 4.81	(27.60 - 79.65)
Sweden	8.98 ± 1.00	(2.31 - 18.44)	8.95 ± 0.99	(2.30 - 18.11)
UK	NA	NA	19.52 ± 2.81	(3.50 - 45.00)

Table S2. Local percentage flower cover in field boundaries and semi-natural habitats in 2011 and 2012. Means ± SE (min - max) are noted.

	2011		2012	
Field boundaries				
Germany	0.11 ± 0.05	(<0.01 - 0.85)	0.26 ± 0.06	(<0.01 - 0.85)
Netherlands	2.64 ± 1.30	(0.04 – 20.84)	2.98 ± 2.09	(0.11 – 34.02)
Sweden	3.22 ± 1.03	(0.07 - 13.37)	1.19 ± 0.27	(0.10 – 3.26)
UK	NA	NA	4.65 ± 0.77	(1.20 - 10.11)
Semi-natural habitats				
Germany	0.18 ± 0.03	(0.04 - 0.49)	0.21 ± 0.03	(0.08 - 0.43)
Netherlands	0.98 ± 0.33	(0.08 – 5.60)	0.41 ± 0.11	(0.07 – 1.56)
Serbia	1.32 ± 0.44	(0.29 - 7.22)	0.35 ± 0.14	(0.06 - 2.20)
Spain	2.27 ± 0.27	(0.57 - 5.29)	1.51 ± 0.12	(0.55 - 2.40)
Sweden	2.49 ± 0.43	(<0.01 - 6.54)	1.84 ± 0.44	(<0.01 - 6.94)
UK	NA	NA	5.18 ± 1.00	(1.18 - 13.84)

Table S3. Results of mixed effect models with *z*-transformed variables relating densities of bumblebees, solitary bees, honeybees and hoverflies in MFC fields, field boundaries and semi-natural habitats to the predictors cover of the mass-flowering crop in a 1 km radius (MFC), cover of semi-natural habitats in a 1 km radius (SNH) and local flower cover (FC). Model estimate (*ß*) and 95% confidence intervals (CIs) are reported. Only significant main effects and interactions are shown.

	**MFC fields**		**Field boundaries**		**Semi-natural habitats**	
	***ß* (95% CIs)**	***P***	***ß* (95% CIs)**	***P***	***ß* (95% CIs)**	***P***
Bumblebees						
MFC	−0.37 (−0.54, −0.20)	<0.001	−0.35 (−0.51, −0.18)	<0.001	−0.16 (−0.31, −0.01)	0.033
SNH	−	−	−0.17 (−0.35, −0.01)	0.048	−	−
FC	−	−	0.25 (0.08, 0.41)	0.004	−	−
MFC:SNH	−	−	−0.20 (−0.37, −0.03)	0.022	−	−
Solitary bees						
MFC	−0.21 (−0.38, –0.04)	0.020	−	−	−	−
FC	−	−	0.35 (0.18, 0.53)	<0.001	0.28 (0.13, 0.42)	<0.001
Honeybees						
MFC	−0.26 (−0.44, –0.09)	0.003	−	−	0.17 (0.02, 0.32)	0.024
FC	−	−	0.25 (0.07, 0.44)	0.007	−	−
Hoverflies						
MFC	−0.19 (−0.37, –0.02)	0.033	−	−	−	−
FC	−	−	0.46 (0.29, 0.62)	<0.001	0.35 (0.21, 0.49)	<0.001
